# Biomechanical characteristics and therapeutic effects of Traditional Chinese Medicine manual therapy in adolescent idiopathic scoliosis: study protocol for a randomized controlled trial

**DOI:** 10.3389/fmed.2026.1840337

**Published:** 2026-05-14

**Authors:** Mengying Rong, Shifang Fu, Longsheng Ren, Lei Bian, Zhenjie Yang, Qian Liu, Yuetong Li, Xiaoyu Zhi, Yu Wang, Yijia Liu, Yanguo Wang

**Affiliations:** 1 Tianjin University of Traditional Chinese Medicine, Tianjin, China; 2 The Second Affiliated Hospital of Tianjin University of Traditional Chinese Medicine, Tianjin, China; 3 Shandong Provincial Hospital Affiliated to Shandong First Medical University, Jinan, Shandong, China

**Keywords:** adolescent idiopathic scoliosis, biomechanics, manual therapy, Schroth method, Traditional Chinese Medicine

## Abstract

**Background:**

Adolescent idiopathic scoliosis (AIS) is a three-dimensional spinal deformity that progresses with age, making effective early intervention crucial to avoid surgical treatment. Traditional Chinese Medicine (TCM) Manual therapy (MT) offers the advantage of relaxing muscles and correcting biomechanical imbalances, thereby assisting patients in performing postural correction exercises under proper biomechanical alignment. However, evidence supporting the use of TCMMT for AIS remains limited.

**Objective:**

This study aims to elucidate the biomechanical changes in AIS patients by comparing with healthy individuals, and investigate the clinical efficacy and biomechanical mechanisms of combining TCMMT with Schroth method in the treatment of AIS.

**Methods:**

This study is a randomized controlled trial. Sixty eligible AIS patients will be randomly allocated in a 1:1 ratio to either the experimental group (EG) or the control group. All patients will undergo Schroth method for 60 min, five times per week. The EG will additionally receive TCMMT for 20 min, three times per week. The treatment course will be 8 weeks. Furthermore, fifteen age-matched healthy volunteers will be included. The primary outcome measure is the Cobb angle assessed before and after treatment. Secondary outcome measures include plantar pressure and 3D gait parameters, surface electromyography and muscular temperature. Adverse events will be recorded throughout the trial. All patients randomly allocated in this study will be included in the intention-to-treat analysis.

**Conclusion:**

This study will provide multi-dimensional evidence for the efficacy of TCMMT as an adjunctive treatment for AIS, advance the development of manual therapy within the field of rehabilitation, and facilitate its application in clinical decision which making by both physicians and patients.

**Study protocol registration:**

http://itmctr.ccebtcm.org.cn, identifier ITMCTR2025002392.

## 1 Introduction

Scoliosis is defined as a lateral curvature of the spine greater than 10 degrees, accompanied by vertebral rotation, with Adolescent Idiopathic Scoliosis (AIS) being the most prevalent type (1). The incidence of AIS is approximately 2 to 4% and shows a consistently increasing trend (2, 3). While AIS can progress during growth and cause cosmetic issues, it is usually asymptomatic and easily overlooked. This neglect can lead to the progression of spinal deformity and overall biomechanical imbalance, resulting in decreased trunk stability and abnormal gait (4–6), which seriously jeopardizes the physical and mental health of adolescents. Therefore, seeking safe and effective early treatment options is of significant importance.

The Society on Scoliosis Orthopedic and Rehabilitation Treatment Recommends Physiotherapeutic Scoliosis-Specific Exercises (PSSE) as an important intervention to halt or slow curve progression (1). The Schroth method is one of the most common PSSE interventions. This method aims to achieve a three-dimensional correction of the patient’s specific curve pattern in daily life by integrating sensorimotor, postural, and corrective breathing exercises (7–9). The Schroth method has been shown to improve posture, alleviate pain, strengthen core muscles, and may contribute to slowing curve progression and reducing the need for surgery. However, the Schroth method requires patients to perform corrective postures under proper biomechanical alignment, but many patients cannot achieve or maintain the postures due to muscle imbalances and joint stiffness. Therefore, its efficacy is highly dependent on patient compliance and varies considerably among individuals (10–13).

According to Traditional Chinese Medicine (TCM) theory, musculoskeletal imbalance is a key etiological factor in AIS (14). As a TCM therapy, TCM manual therapy (MT) is regarded as a safe and effective therapeutic approach and widely used in the treatment of AIS (15–17). By reducing pain, decreasing spinal stiffness, and restoring biomechanical dysfunction in affected segments, TCMMT may enhance patients’ comfort and compliance with daily exercise. In addition, TCMMT allows patients to perform the effective Schroth method under corrected biomechanical alignment, which complements the benefits of exercise therapy (16, 18).

The lower limbs, pelvis and spine are interconnected. Existing studies have widely reported that AIS patients typically exhibit biomechanical abnormalities in both the spine and lower limbs, such as asymmetric muscle activation, temperature differences in muscle activity, gait abnormalities and abnormal plantar pressure distribution, which further compromise spinal stability by disrupting the biomechanical balance between spine and lower limbs (6, 19–21). Understanding these biomechanical features is important for developing personalized rehabilitation strategies for AIS. However, the biomechanical changes of AIS patients are still controversial (22–24). The impact of TCMMT on biomechanics also has not been fully elucidated, and the quality of relevant studies still needs improvement (25, 26).

Thermography is a useful and noninvasive method of assessing muscular tension imbalance in the course of scoliosis (27). Combining with the plantar pressure, 3D gait analysis, and surface electromyography (sEMG) enables a comprehensive evaluation of the spine-limb biomechanical patterns during standing and walking in patients. Consequently, this study will employ the Cobb angle, the gold standard for scoliosis assessment (28), and integrate plantar pressure measurements, 3D gait analysis, surface electromyography (sEMG) and infrared thermography (IRT) to evaluate biomechanical changes. The study aims to elucidate the characteristics of biomechanical changes in AIS during standing and walking by comparing with healthy individuals at baseline, and provide evidence-based support for the efficacy and safety of combined TCMMT and Schroth method therapy by comparing baseline and post-treatment data of AIS.

## 2 Materials and methods

### 2.1 Study design and settings

**Figure 1:**
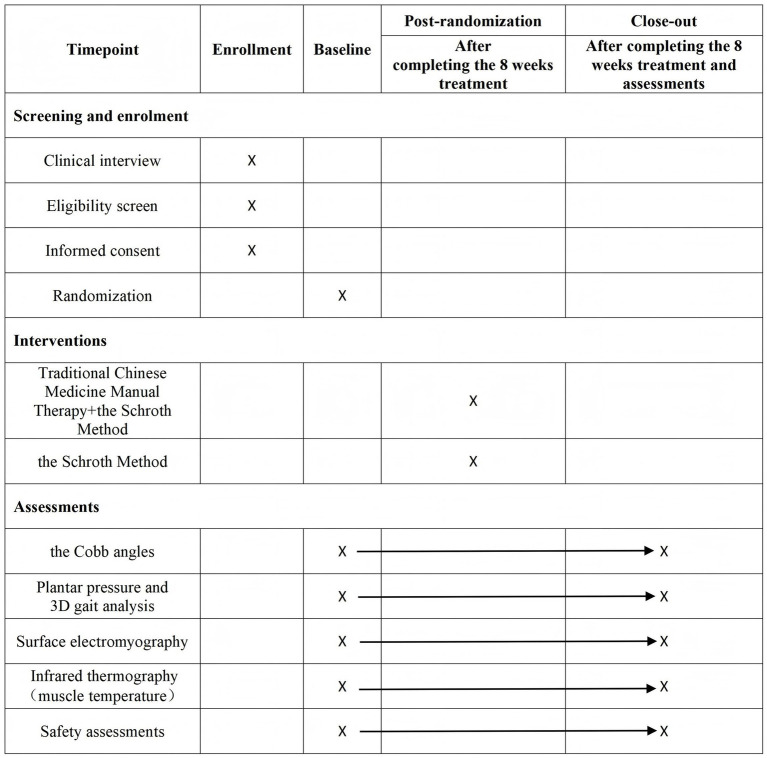
The SPIRIT schedule of participant timeline.

**Figure 2:**
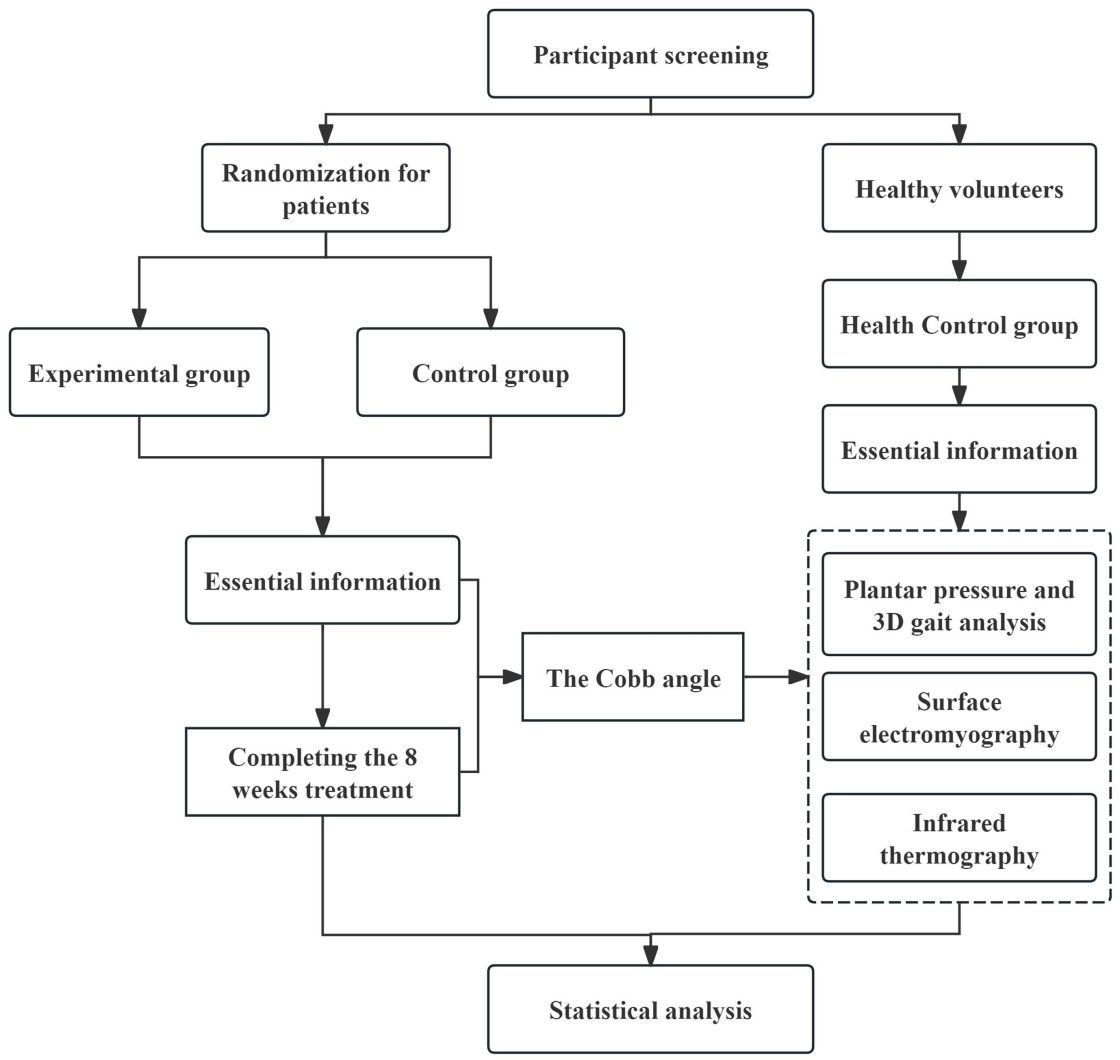
The flow chart of the trial procedure.

This study is a prospective randomized controlled trial. This study compares the biomechanical patterns between AIS patients and healthy individuals during standing and walking using technologies such as plantar pressure measurements, 3D gait analysis, sEMG, and IRT, and observes changes in patients before and after treatment. The research will be conducted at the Rehabilitation Medicine Department of the Second Affiliated Hospital of Tianjin University of Traditional Chinese Medicine. A total of 60 patients will be enrolled, along with 15 healthy individuals of similar age. Patients in the control group (CG) will perform the Schroth method for 60 min, five times per week. The experimental group (EG) will receive 20-min TCMMT three times per week in addition to the Schroth method. The total treatment duration for both groups is 8 weeks. The healthy control (HC) will receive no intervention. All patients will be assessed for Cobb angle, 3D gait parameters, sEMG and IRT before and after treatment. The healthy individuals will receive all assessments except the Cobb angle measurement only upon enrollment. The SPIRIT schedule of participant timeline is shown in Figure 1 and the flow chart of the trial procedure is presented in Figure 2.

### 2.2 Study participants

#### 2.2.1 Recruitment strategy

We will screen and recruit participants from December 2025 to August 2026 at the second affiliated hospital of Tianjin University of Traditional Chinese Medicine. Our researchers will be systematically trained and fully informed of the trial procedures. Informed consent will be obtained from participants before enrollment. Participation will be entirely voluntary, and participants will have the right to withdraw from the trial at any time without penalty.

#### 2.2.2 Inclusion criteria

1. Diagnostic criteria for idiopathic scoliosis (1).
2. Ages 8 to 15 years, skeletal immaturity (defined as a Risser grade ≤2) (29, 30).
3. Major curve Cobb angle 10° ~ 40°.
4. No obvious deformities in the lower limbs and feet.
5. Can understand and follow instructions to walk independently.
6. Can understand and voluntarily participate in, and sign the informed consent form.

#### 2.2.3 Inclusion criteria for the HC

1. Ages 8 to 15 years.
2. Can understand and voluntarily participate in, and sign the informed consent form.

#### 2.2.4 Exclusion criteria

1. Major curve Cobb angle>40°, the patient has used brace.
2. Non-specific scoliosis.
3. Abnormalities in growth and development, as well as neurological, muscular, skeletal, and mental functions.
4. The patient and guardian refuse or are unable to cooperate with the completion of the assessment.
5. Participating in other relevant clinical studies.

#### 2.2.5 Termination criteria

The termination criteria are as follows: (1) Participants’ voluntary withdrawal, such as revoking informed consent; (2) Participants whose condition progressively worsens during the trial, and for whom the physician determines discontinuation of the clinical trial is necessary. To protect the subject, they should withdraw from the trial; (3) During the trial, participants demonstrate poor compliance, fail to undergo intervention as specified; and (4) Participants are experiencing serious adverse events.

#### 2.2.6 Sample size consideration

According to previous research (31), the Cobb angle was 10.87 ± 3.60°in EG after treatment, compared to 14.97 ± 5.01°in CG. Using the PASS 2021 software for calculation, with a sample size ratio set at 1:1, *α* = 0.05 (two-sided test), and a statistical power (1−*β*) of 0.90, while controlling the attrition rate within 15%, it is determined that 30 patients need to be recruited for each group. Therefore, this study will require the inclusion of a total of 60 patients. In addition, we will recruit 15 healthy individuals of the same age. They will compare with patients at baseline to evaluate biomechanical changes of AIS.

#### 2.2.7 Randomization and blinding

Eligible patients will be randomly assigned to either the EG or the CG in a 1:1 ratio. The randomization sequence will be generated by a statistician not involved in outcome assessment using SPSS 27.0, and placed in opaque, sealed envelopes by a researcher not involved in trial intervention, assessment and data analysis. After an eligible patient is enrolled, the physician will obtain the allocation result by opening the corresponding envelope. Throughout the trial, the randomization sequence will remain inaccessible to other researchers. While blinding of the physicians and patients is not feasible, outcome assessors and the data statistician will remain blinded to group allocation.

### 2.3 Interventions

Patients in both groups will receive the Schroth method for 60 min, 5 times per week. In addition to the Schroth method, the EG will receive TCMMT for 20 min, 3 times per week. The total treatment duration for both groups will be 8 weeks. The HC will receive no treatment.

#### 2.3.1 The Schroth method

The Schroth method will include daily postural training (sitting, lying, and standing), personalized breathing exercises, simplified three-dimensional correction, and strengthening exercises (32). Specific operational procedures are detailed in Table 1.

**Table 1:** Specific operational procedures of the Schroth method.

Operation item	Specific operational procedures
Daily postural training (sitting, lying, and standing)	The patients will be instructed to shift their body weight toward the concave side, abduct the arm on the concave side slightly above shoulder level, rest the elbow on a horizontal bar, and place the hand behind the head. During the side-lying position, the patients should lie on the concave side of the curvature, allowing the spinal convexity to shift toward the midline under the influence of gravity.
Personalized breathing exercises	The patients will be instructed to straighten their torso and perform deep breathing. The practitioner will apply gentle pressure with the palm of the hand on the concave side of the spinal curvature. The patients will be asked to take a deep, diaphragmatic breath, and during inhalation, to slowly expand the area under the practitioner’s hand, pushing against the pressure.
Simplified three-dimensional correction	Including core components such as midline shift, pelvic adjustment, rotational breathing, and muscular contraction force generation. Corrective movements will be integrated into daily postures and activities.
Strengthening exercises	The patients will sit facing the wall bars with the concave side of the spine oriented toward them. To stabilize the trunk, the thighs will be abducted and externally rotated. The hand on the convex side will be placed on the ipsilateral shoulder to perform shoulder counter-traction. The hand on the concave side will be passed through a horizontal bar at shoulder height and will grip a bar at waist level, thereby opening the thoracic concave side.

#### 2.3.2 Traditional Chinese Medicine manual therapy

The proprietary spinal balance-regulating TCMMT developed by the Rehabilitation Department will be applied to soft tissue relaxation and joint adjustment. First, the dynamic balance of the spine will require adjustment, including relaxing the bilateral paraspinal muscles, releasing the persistently contracted muscles on the concave side and the tense muscles on the convex side. Following this, Chiropractic spinal manipulations will be performed at the spinal levels that do not maintain correct alignment, including the cervical, thoracic, lumbar vertebrae, or pelvis. The specific operational methods and durations are detailed in Table 2.

**Table 2:** Specific operational procedures of the Traditional Chinese Medicine manual therapy.

Operation item	Specific operational procedures
Regulating the dynamic balance of the spine	
Relaxing the bilateral paraspinal muscles	The practitioner will apply the palm of the hand to the spine and the bilateral paravertebral muscles, and move in a unidirectional straight or arc-shaped line parallel to the spinal column to relax the paravertebral muscles, with each session lasting 4 min. The practitioner will hold the four fingers together with slight flexion and naturally abduct the thumb slightly. Using the dorsal metacarpophalangeal joints of the little, ring, and middle fingers to contact the paraspinal muscles, the practitioner will perform the manual therapy through a continuous external rotation movement of the wrist joint, with each session lasting 4 min.
Releasing the persistently contracted muscles on the concave side	The practitioner will use the thenar eminence or the heel of the palm to apply focused pressure on the paraspinal muscles of the convex side. A rotary movement will be performed using the wrist joint, with moderate, even force and a steady rhythm. Each session will last 5 min.
Releasing the tense muscles on the convex side	The practitioner will use the thumb(s) of one or both hands to apply deep pressure to the paraspinal muscles. Once a sensation of soreness or pain is perceived under the finger(s), a back-and-forth plucking motion, similar to plucking a string, will be performed. Each session will last 5 min.
Chiropractic spinal manipulations: Adjust the static balance of the spine at the spinal levels that does not maintain correct alignment, include the cervical, thoracic, lumbar vertebrae or pelvis. Each session will last 2 min.	
Cervical spine adjustment	The patient will lie in a supine position. The practitioner, positioned at the head of the patient, will firmly grasp the posterior aspect of the patient’s cervical spine. A manual adjustment will be performed while maintaining traction.
Thoracic spine adjustment	The patient will lie in a prone position. Taking a right thoracic curve as an example, the patient’s head will be tilted toward the convex side. The practitioner will apply pressure with the palm along the thoracic spine. Using the thumb, the spinous processes of the thoracic vertebrae will be sequentially pushed, focusing particularly on the apical vertebra of the curve.
Lumbar spine adjustment	The patient will lie in a lateral decubitus position. Hip and knee flexion will be used to position the axis of spinal rotation at the L3-L4 level. The practitioner will apply pressure with the thumb against the lumbar spinous processes and perform adjustments sequentially.
Pelvic adjustment	For anterior or posterior pelvic tilt, the practitioner will brace against one ischial tuberosity and the superior aspect of the contralateral ilium, applying an impulse in opposite directions.

### 2.4 Outcomes measures

Collect general information of participants, including name, gender distribution, age, risser grade distribution, height, weight, and medical history.

#### 2.4.1 Primary outcome measure

All AIS patients will require the standing frontal radiograph. The Cobb angles will be measured by two blinded and independent radiologists before and after the treatment.

#### 2.4.2 Secondary outcome measures

All participants will be assessed for plantar pressure, 3D gait parameters, sEMG and muscle temperature at baseline upon enrollment. AIS patients will receive a second assessment after completing the treatment. All participants will acclimatize to the testing environment for 10 min before data collection. The indoor conditions will be maintained as follows: a quiet setting with minimal air movement, absence of strong light or direct sunlight, an ambient temperature of 26 °C, and a relative humidity of 40–60%. Air conditioning vents or outlets will be positioned away from both the participants and the equipment to prevent direct airflow or radiant heat from interfering with the participants or the infrared thermal camera.

##### 2.4.2.1 Plantar pressure and 3D gait parameters

Plantar pressure measurement and 3D gait analysis provide critical information on kinematic, kinetic, and spatiotemporal parameters (33, 34). The data extracted from these analyses can be used for gait abnormality analysis, sports performance evaluation, balance control improvement, and the detection of functional impairments, among other applications (35, 36).

In this study, the biomechanics of plantar pressure distribution and standing balance status will be evaluated by using the FeetMapping plate-type plantar pressure measurement system. This system will be used in conjunction with the RealGait 3D gait and motion analysis system to collect spatiotemporal gait parameters and joint range of motion during walking.

##### 2.4.2.2 Surface electromyography

The activities of the bilateral paraspinal muscles (at cervical, thoracic, and lumbar levels), quadriceps, semitendinosus, tibialis anterior, and lateral gastrocnemius will be measured by a 16-channel MyoMove-EOW sEMG system to systematically evaluate the overall muscle activation patterns of the spine and lower limbs during standing and walking states. Recordings will be obtained during both quiet standing and walking. Muscle recruitment will be assessed by analyzing the amplitude variation of the bioelectrical signals, with the root mean square value recorded for quantitative analysis.

##### 2.4.2.3 Muscular temperature

The study will utilize the IRT to capture thermal images of the nape and back region of participants. Temperature changes in the bilateral paraspinal muscles (at cervical, thoracic, and lumbar levels) will be recorded.

##### 2.4.2.4 Adverse events

During the treatment period, any newly emerged symptom will be documented as adverse events, including but not limited to aggravated pain, muscle spasms, dizziness, or headache. The physician will record in detail the severity, time of onset, duration, management measures, and course of each event. All AEs will be truthfully recorded on an AE form, capturing the time of occurrence, severity, duration, actions taken, and outcome. According to grading criteria for AEs, the clinical trial of this case should be stopped according to the judgment of the doctor and given compensation to those who suffer harm from trial participation when serious adverse events occur.

### 2.5 Data management and analysis

#### 2.5.1 Data management

Data entry personnel and outcome assessors will receive standardized training in data management procedures. All participants’ information will be recorded in original case report forms. Two individuals will independently enter the data into Excel spreadsheets and cross-verify the entries for accuracy. In cases of improper data recording, the data manager will make necessary corrections. All paper documents related to the study will be securely archived, while electronic data will be stored on password-protected computers to ensure security of the data. All materials must be retained for at least 5 years after publication. If readers have questions regarding the published study findings, they may contact the corresponding author to request access to the original dataset. Personal information of participants will be kept confidential.

#### 2.5.2 Data analysis

All statistical analysis will be performed by an independent statistician (blinded to group allocation) using SPSS 27.0. A *p*-value of <0.05 will be considered statistically significant. Continuous data will be summarized as mean ± standard deviation (M ± SD) or median with interquartile range. Categorical variables will be presented as frequencies or percentages.

This study will employ an intention-to-treat analysis, including all participants who were randomized and received at least one intervention session. Outcomes will be analyzed using two-sample *t*-tests or paired *t*-tests. If normality assumptions cannot be met, non-parametric tests will be used. For categorical variables, chi-square (χ^2^) tests will be performed. Inter-rater reliability will be assessed using the intraclass correlation coefficient. For case data with missing values, multiple imputation will be used to address the gaps.

### 2.6 Quality control

The study protocol has been approved by the Second Affiliated Hospital of Tianjin University of Traditional Chinese Medicine Ethics Committee. To ensure the quality of the research and the safety of participants, all researchers involved in the study will undergo unified and standardized training. TCMMT interventions will be performed by attending physicians with over 5 years of experience. Training and instructional videos will be recorded by senior chief physicians from the Department of Rehabilitation, and training and assessment protocols will be developed. Only researchers who pass the assessment will be qualified to perform the interventions. The study will establish a clear testing process and develop standardized operational procedures to strictly control the quality of the observed indicators and ensure the safety of participants. Participants will be enrolled strictly in accordance with the diagnostic, inclusion, and exclusion criteria. If a participant fails to complete the treatment and observation period as required by the protocol, researchers will attempt to contact them to inquire about the reasons and complete the assessment items. All data will be objectively collected using a combination of paper records and electronic documentation. All issues encountered during the trial will be accurately recorded. Outcome assessors and statisticians will be strictly blinded.

## 3 Discussion

Scoliosis may lead to psychological issues, pain, respiratory complications, and functional limitations (37–40). Due to its complex three-dimensional spinal curvature and potential for progression, it poses a significant challenge in musculoskeletal rehabilitation (41). Therefore, early intervention during the adolescent developmental period is essential to prevent disease progression.

Numerous studies have demonstrated that Schroth exercise has positive effects on enhancing back muscle strength, improving respiratory function, slowing curve progression, reducing the Cobb angle, and lowering surgical rates (13, 42, 43). However, the efficacy of this method relies heavily on patients’ long-term compliance, and the postural correction training must be performed under correct biomechanical alignment conditions. TCMMT demonstrates notable advantages in this regard (16, 18). MT can enhance improvement in the Cobb angle, as well as alleviate pain, psychological distress, and disability. Furthermore, studies indicate that MT should be considered as a complementary and alternative therapy for the effective management of AIS patients (44, 45). However, the current evidence supporting MT for AIS remains insufficient, especially for TCMMT (18, 24, 46). This gap stems from both the unclear mechanisms underlying its therapeutic benefits and the generally low methodological quality of existing research. Moreover, the biomechanical alteration mechanisms in AIS are still controversial, and there is a notable lack of multimodal quantitative analysis characterizing these biomechanical changes in AIS.

This study innovatively integrates plantar pressure measurement, 3D gait analysis, sEMG, and IRT to establish a multimodal quantitative assessment framework. This system comprehensively captures the biomechanical characteristics of AIS patients during both static standing and dynamic walking. The approach synergistically complements and validates findings across multiple dimensions, including postural support, movement patterns, muscle activation, and thermal asymmetry, thereby significantly enhancing the objectivity and accuracy of the observed metrics. This protocol will not only enable an in-depth exploration of the biomechanical mechanisms underlying postural and gait abnormalities in AIS patients but also provide a precise and individualized basis for evaluating the therapeutic effects of TCMMT. It will hold substantial value for clinical research and possess promising application prospects.

The study will be conducted in strict adherence to the protocol, and all researchers will receive unified, standardized training to ensure compliance with regulated trial procedures. Based on these rigorous methodological designs, we believe that the results of this trial will provide new evidence regarding the role of manual therapy in AIS and make a substantive contribution to the current evidence base for the physical therapy of scoliosis.

This trial shares similar limitations with previous studies. Due to the nature of the interventions, neither the physicians nor the patients could be blinded, which may introduce bias. However, the primary objective of this study is to evaluate the additive effect of TCMMT within the combined regimen, rather than to independently validate the effectiveness of exercise therapy. This study will focus on assessing the corrective effect of combining TCMMT with the Schroth method on biomechanical imbalance by integrating multiple quantitative and objective evaluation metrics.

## 4 Conclusion

This study will comprehensively evaluate the characteristics of biomechanical changes in AIS during standing and walking by comparing with healthy individuals, and the effects of combining TCMMT with the Schroth method in regulating overall biomechanics in the treatment of AIS. The findings are expected to provide evidence supporting the integration of traditional Chinese and Western rehabilitation approaches. Furthermore, the results may facilitate the application of such combined strategies in clinical decision-making by both physicians and patients, offering a new therapeutic option for individuals with AIS.
